# Parameters Influencing the Emission of Ultrafine Particles during 3D Printing

**DOI:** 10.3390/ijerph182111670

**Published:** 2021-11-06

**Authors:** Radomír Chýlek, Libor Kudela, Jiří Pospíšil, Ladislav Šnajdárek

**Affiliations:** Energy Institute, Faculty of Mechanical Engineering, Brno University of Technology—VUT Brno, Technicka 2896/2, 61669 Brno, Czech Republic; Libor.Kudela@vutbr.cz (L.K.); pospisil.j@fme.vutbr.cz (J.P.); snajdarek@fme.vutbr.cz (L.Š.)

**Keywords:** 3D printing, fine particles, emissions, thermoplastics, size distribution, air quality

## Abstract

This paper presents a complex and extensive experimental evaluation of fine particle emissions released by an FDM 3D printer for four of the most common printing materials (ABS, PLA, PET-G, and TPU). These thermoplastic filaments were examined at three printing temperatures within their recommended range. In addition, these measurements were extended using various types of printing nozzles, which influenced the emissions considerably. This research is based on more than a hundred individual measurements for which a standardized printing method was developed. The study presents information about differences between particular printing conditions in terms of the amount of fine particles emitted as well as the particle size distributions during printing periods. This expands existing knowledge about the emission of ultrafine particles during 3D printing, and it can help reduce the emissions of these devices to achieve cleaner and safer 3D printer operations.

## 1. Introduction

During the COVID-19 pandemic, 3D printing (or Additive Manufacturing—AM) demonstrated its great potential and the flexibility to fight the disease. 3D printers, which have already become a typical part of labs, classrooms, workshops, and homes, became firmly established in public awareness. Mainly during the first months of the COVID-19 outbreak, the complete lack of resources for patients and caregivers was very quickly addressed by both professional and hobbyist 3D printing providers, designers, and makers. These smaller, but well-distributed, communities of decentralized manufacturers played a significant role worldwide by printing protective equipment such as masks, shields, or components of medical devices [1]. But are 3D printers safe for their operators and surroundings? And what should be done to ensure operator safety and health?

Even when a 3D printer operates properly, there is a risk of inhaling hazardous particles or gaseous products during the extrusion of melted thermoplastics. The main cause of this phenomenon is insufficient ventilation: the absence of air filters and air purifiers in the interior occupied with a 3D printer. As a result, these pollutants accumulate and create toxic indoor conditions. Many professional industrial 3D printers are equipped with filtration systems and are usually placed in an air-tight chamber. However, these precautions are not common for regular consumer-level printers. 

There is some recent research on the negative health effects of exposure to 3D printer emissions. For a short exposure of subjects (1 h), no acute health effects were found and most of the measured biochemical responses were typical. A relative increase in exhaled nitric oxide (FeNO) and self-reported odor nuisance was greater when printing with ABS (acrylonitrile butadiene styrene—a common plastic for 3D printing) than with PLA (polylactic acid—another widely used filament material) [2].

Chan et al. [3] found that working more than 40 h per week with 3D printers may result in respiratory-related diagnoses such as asthma or allergic rhinitis. Utilizing a human airways replica, the cumulative tracheobronchial deposition of ultrafine particles from 3D printing was estimated to be 1%–13%, depending on the particle size [4]. Farcas et al. [5] exposed human small epithelial cells to 3D printer emissions while operating with ABS and polycarbonate. After the exposure, both polycarbonate and ABS emissions induced dose-dependent cytotoxicity, oxidative stress, and other toxicological effects.

The problem of fine particle (FPs) emissions from 3D printing was first addressed by Stephens et al. [6], who reported the measurements of FPs during the operation of several printers with two types of thermoplastic filaments. In their work, the total emission rates and size-resolved rates were estimated. Soon after, more studies arrived at similar estimations. Particle emissions from ABS and PLA filaments were evaluated and compared across the literature in a meta-analysis study by Byrley et al. [7]. It was also found that the emission of FPs is dependent not only on the filament type but also on its color and filament additives [8]. 

The number of usable thermoplastic filaments is constantly growing, and there is an effort to improve the variety of properties of these materials. Nowadays, we can come across various composite filaments with the addition of metals or carbon nanotubes. However, caution is needed and some mechanisms should be put in place to control the emissions of these filaments, as they can release hazardous nanoparticles [9]. PET-G (polyethylene terephthalate glycol-modified) is also a relatively new material, but it is becoming very popular among consumers even though the FP emissions of this material seem poorly described in the current literature [10].

Generally, the distribution of aerosol behaves differently when the printer is located inside an experimental (or safety) chamber or is under real-use indoor conditions. Floyd et al. [11] used eight filament types to characterize FP and VOC (volatile organic compound) emissions. He reported that during the initial printing and pre-heating period a relatively large burst of FPs occurred, followed by a decline in FP concentration. The mechanisms regulating the nucleation of new particles and their subsequent coagulation and growth are poorly understood. It depends, for example, on the conditions and dilution of the environment as well as the nature of the aerosol particles [12]. The rapidly growing particle distributions are more observable inside enclosed chambers. The so-called “banana-shaped” distributions were found in several emission chamber studies, and not only in those related to 3D printing [13].

Other studies have focused more on the gaseous emissions of the 3D printers. Kim et al. [14] found that, except for FPs, several aldehydes, phthalates, and VOCs such as toluene and ethylbenzene were emitted. Azimi et al. [15] tested the FP and VOC emissions of five different printers and nine filaments in a closed test chamber. Their study allowed a more detailed comparison of the filaments and identified the emission of additional VOCs, such as styrene and caprolactam. 

Some researchers have studied the FP emission of thermoplastic filaments while heated by a different source than the 3D printer, such as by a 3D pen [16]. A 3D pen is a handheld device, so its users remain constantly close to the pollutant source during its use, which could result in a much higher exposure compared to the 3D printer. Another laboratory heat source is a thermogravimetric analyzer. It was used to study the thermal stability and decomposition temperatures of thermoplastics along with their VOC emission [17]. In our previous study [18], we used a similar technique to model the behavior of thermoplastics during printing by heating them to their printing temperature inside the thermogravimetric analyzer. It was found that the thermogravimetric analysis can predict the FP emission of various types of thermoplastics during 3D printing. A similar technique was later used for the analysis of the ABS filament by Sittichompoo [19].

Previous studies of the printing parameters have shown that the nozzle temperature during extrusion is a critical parameter for the reduction of FPs. According to Deng et al. [20], a higher nozzle temperature leads to higher FP emissions. A similar conclusion was presented by Stabile et al. [21] who related higher emissions to the higher material decomposition and higher vapor pressure of organic material occurring at higher temperatures, which leads to an increase in particle nucleation. Unfortunately, lowering the temperature is not always possible because of reliability and print quality, which are associated with specific optimal printing temperatures for the material. On top of this, it is often the higher temperatures that will allow the resulting print to have advantageous mechanical properties [22].

In this work, we did an extensive experimental analysis of FP emissions during 3D printing using the four most sought-after filament materials (ABS, PLA, PETG, and TPU—thermoplastic polyurethane), according to Google Trends (as of 03/2021). We printed every material at three nozzle temperatures, at the lower and the upper limit of recommended printing temperatures (specified by the manufacturer), and at its middle value. To our knowledge, we are the first to also test various extruder nozzle diameters. All combinations of materials and temperatures were printed with three printing nozzles with different extruder diameters. Every measurement was repeated three times, resulting in more than a hundred individual samples analyzed. Our intent was to make a comprehensive comparison of all the mentioned print configurations, to recommend ideal printing parameters for reducing fine particle emissions. 

## 2. Materials and Methods

### 2.1. Chamber Setup

Experiments were performed in an airtight composite test chamber with a volume of 0.3 m^3^. This allowed us to measure the particle emission regardless of the background particle concentration. Inside the chamber, there were no air filters, air purifiers or other forms of particle-capturing devices. Two ventilators (one as a part of the print head, the other as an auxiliary component) were present to create a homogenous distribution of particles inside the chamber. The average temperatures and humidity inside the chamber were continuously monitored. The temperature inside the chamber during the printing was between 25 °C and 35 °C and relative humidity was around 50%. On the top of the test chamber a conductive stainless-steel tube with a diameter of 2 mm was installed to serve as an aerosol offtake. The inlet of the tube was placed approximately 10 cm from a moving print head. The volumetric flow rate of exhausted aerosol was at a constant rate of 300 cm^3^/min. The same amount of particle-free air was supplied to the chamber through the HEPA filter. The air exchange rate (AER) was kept at a relatively low level (0.06 h^−1^).

### 2.2. Particle Sampling

**Figure 1:**
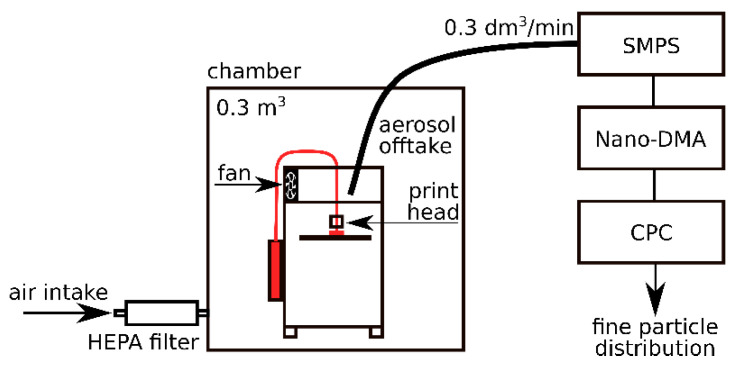
Diagram of the experimental setup.

Aerosol was exhausted directly from the chamber into the TSI scanning mobility particle sizer unit (SMPS, TSI Inc., Shoreview, MN; model 3080). Instead of the standard Long-DMA, the Nano-DMA (TSI Inc., Shoreview, MN; Differential Mobility Analyzer model 3085) was used. Although it has a generally narrower working range for the particle diameters, it covers the measured range better, with a range of 2 to 150 nm. A butanol-based Condensation Particle Counter (CPC, TSI Inc., Shoreview, MN; model 3775) was used for detecting the amount of particles, which provided their particle number distribution. A diagram of the experimental setup is shown in Figure 1.

Every print configuration was printed three times and different sampling times were used for every individual print. Short sampling intervals are more prompt to noise but provide a higher temporal resolution. Due to potential drawbacks in the precision of fast scans, the longer scans (60 s and 120 s) were performed in the second and third runs. 

The particle concentration was monitored before printing and the measurement did not begin until the background concentration inside the chamber fell below a negligible 200 particles/cm^3^. Particle measurement continued even after the printing, in order to capture data for the estimations of the total exponential decay rate of the particles (loss factor K).

### 2.3. Filament Materials and 3D Printer

The data exploration tool Google Trends was used to identify the most popular filament materials worldwide. The most sought-after group consisted of four materials mentioned below, all with a significant lead in searches over the others. Acrylonitrile butadiene styrene (ABS Yellow, Ultimaker, Utrecht, The Netherlands) is a very popular choice for hard and durable products. Polylactic acid (PLA Extrafill Vertigo Grey, Fillamentum, Hulin, Czech Republic) is easy to print, cheap, and soft biomaterial. Polyethylene terephthalate glycol-modified (PET-G Polylite Teal, Polymaker, Suzhou, China) combines the properties of ABS and PLA. Lastly, thermoplastic polyurethane (TPU95A Red, Ultimaker) is a material with high flexibility, resilience, and chemical resistance. All filaments had a diameter of 2.85 mm.

The 3D printer used for this study was the Ultimaker 3 Extended with original printcores of AA 0.4 mm, AA 0.25 mm, and HardCore 0.6 mm nozzle (3D Solex). Selected printing temperatures represent three steps over the commonly recommended printing temperature range given by the producers. All print configurations were printed without visible defects or inaccuracies. Standard printing profiles from the Ultimaker Cura software were used except for the TPU in combination with a 0.25 mm nozzle. It is not officially supported or recommended to print the TPU with a 0.25 mm nozzle due to the excessive nozzle clogging. For printing TPU with a 0.25 mm nozzle, a modified 0.40 mm nozzle profile with a lower layer width was used. All the printing configurations with the lengths of filaments used and the average mass flow of extruded filament are shown in Table 1.

**Figure 2:**
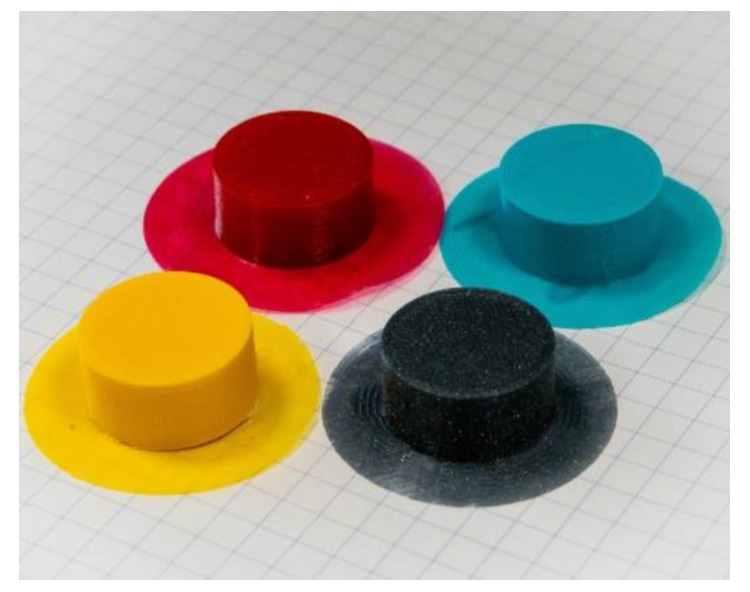
Example of printed samples with a detachable brim structure, yellow—ABS, red—TPU, blue—PET-G, grey—PLA.

Due to the lack of a standardized printing design for the evaluation of emissions of the 3D printer, we created a model. The design is a simple cylinder with default infill, which limits any potential printing errors caused by a complex shape. The cylinder has a diameter of 20 mm and height of 10 mm. To create better adhesion with the build plate of the printer, a detachable structure called a “brim” was printed under the sample (Figure 2). After the print was finished, the chamber was opened, the printed object was removed from the printing bed, and the printing nozzle was cleaned of burnt filament residues.

### 2.4. Statistical Analysis

Every print configuration was independently printed three times, while for SMPS different scan times were used (30, 60, 120 s) and the resulting particle distribution was averaged. The obtained particle number distributions were processed in batches using Python code with the libraries Pandas and Bokeh to process, analyze and visualize the results. All the measured data will be available online: www.github.com/RadomirChylek/3d-printing-emission after publishing (access date 1 November 2021).

To perform the following calculations, it is first necessary to determine the rate at which the particle concentration is decreasing inside the experimental chamber. The particle loss factor is calculated using the exponential decay of total particle concentration (sum of the concentrations within all the measured size bins) that occurs after the printing period. After the 3D printer has finished, it is assumed that there is no other source of particles in the chamber and the particles can only disappear. The loss factor accounts for both the deposition of particles onto the present surfaces as well as the dilution due to inflow of filtered air and outflow of the aerosol into the SMPS:

dC_t_/dt = k_ct_ · C_t_ = (−V_a_ + k_cd_·V/V) · C_t_ (1)

where *C_t_* [particles/cm^3^] is total particle concentration, t [s] is time, V [cm^3^] is the volume of air in the chamber, k_ct_ [1/s] is the total loss factor with respect to total particle concentration, k_cd_ [1/s] is deposition loss factor with respect to total particle concentration, and V_a_ [cm^3^/s] is the outflow of air from and into the chamber.

Another way to evaluate the particle losses is to replace total particle concentration with total particle volume. As shown further below, this approach was more useful in our case.

dV_pt/_dt = k_vt_ · V_pt_ = (−V_a_ + k_vd_·V/V) · V_pt_ (2)

where V_pt_ [nm^3^/cm^3^] is the total volume of particles, k_vd_ [1/s] is deposition loss factor with respect to the total volume of particles, k_vt_ [1/s] is the total loss factor with respect to the total volume of particles.

**Figure 3:**
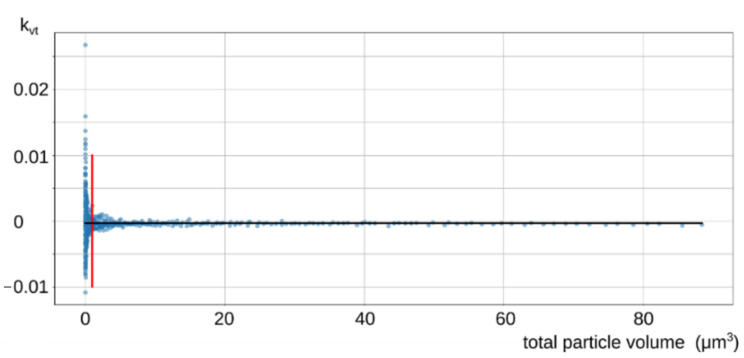
Loss factor with a line splitting the low uncertainty interval.

Individual samples are used to calculate the loss factor using the following numerical formula 3. The uncertainty of the values is higher with lower total concentrations of particles (or lower total volumes of particles). This is visualized in Figure 3. The mean value of the loss factor (black line in Figure 3) is therefore evaluated only from concentrations that are above the low uncertainty threshold (the red line in Figure 3).

k_vt_^i^ = (V_pt_^i+1^ − V_pt_^i^)/(V_pt_^i^ · ∆t) (3)

The particle (number) concentration emission rate (PNER) was evaluated as it is not applicable for our case. Due to the very low air exchange rate (AER—0.06 h^−1^) of our poorly ventilated chamber, the coagulation and aggregation of particles was significantly predominant over the nucleation of new particles, often resulting in negative particle number emission rates (coagulation acts as unaccounted particle loss). This has very low explanatory power about what is happening inside the chamber, while taking the coagulation mechanism into account when calculating loss factors is too challenging. We tested all the particle number emission rate formulas summarized in [7] with similar results. 

As there is currently no consensus on how to standardize emission measurements and the evaluation of 3D printers, we decided to propose an emission rate model which uses the volume of particles instead of their number—a particle volume formation rate. Assuming that the particles are spherical and have a constant mass density, the total particle volume follows the conservation law which limits the description to equation 4. The value of the loss factor in this relationship is the mean value for each studied material.

dV_pt_/dt = k_vt_^averaged^ · V_pt_ + PVFR/V (4)

where PVFR [µm^3^/s] is the particle volume formation rate.

The numerical formula that was used has the following form:

PVFR^i^ = V · (V_pt_^i+1^ − V_pt_^i^)/∆t − V · k_vt_^averaged^ · v_pt_^i^ (5)

The particle volume formation rate can reflect the growth of particles during our measurements under more specific prevailing conditions inside our chamber and we are thus able to create a better comparison of the results.

## 3. Results and Discussion

**Figure 4:**
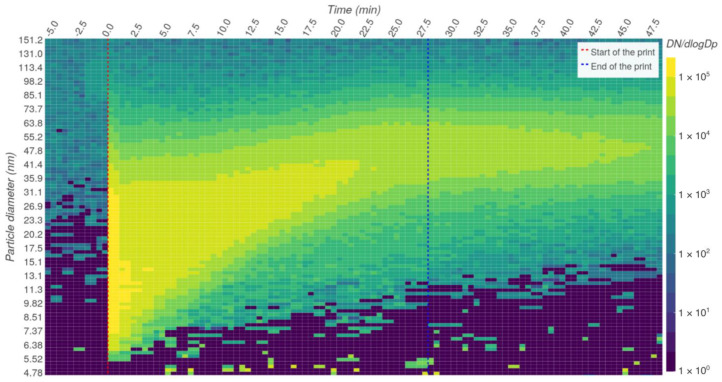
Example of the measured particle distribution.

In the initial preparation period, the chamber was purged with particle-free air to negligible particle concentrations (total particle concentration < 200 particles/cm^3^). This period can be seen in the example in Figure 4, before the vertical red line which shows the start of the print.

**Figure 5:**
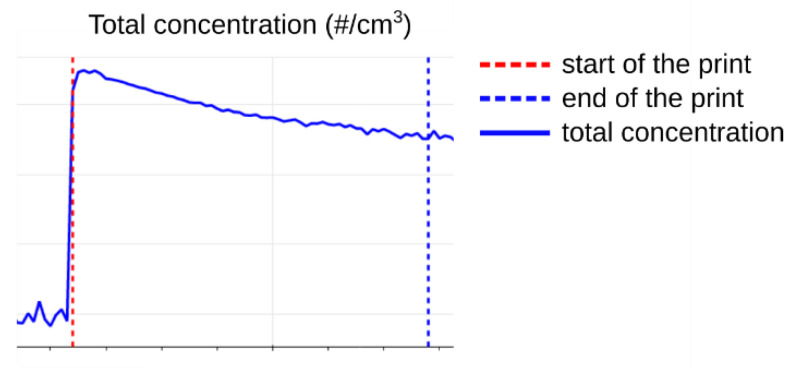
Prevalent progression of total particle concentration during the printing of PET-G.

After that purge was completed, the printing job was sent to the printer, and the printer started to heat the printing nozzle and subsequently initiated the extrusion of the filament. In the first few minutes of the printing, the value of total particle concentration commonly reached its maximum, as shown in the example of printing PET-G at 250 °C with a 0.4 mm nozzle (Figure 5).

**Figure 6:**
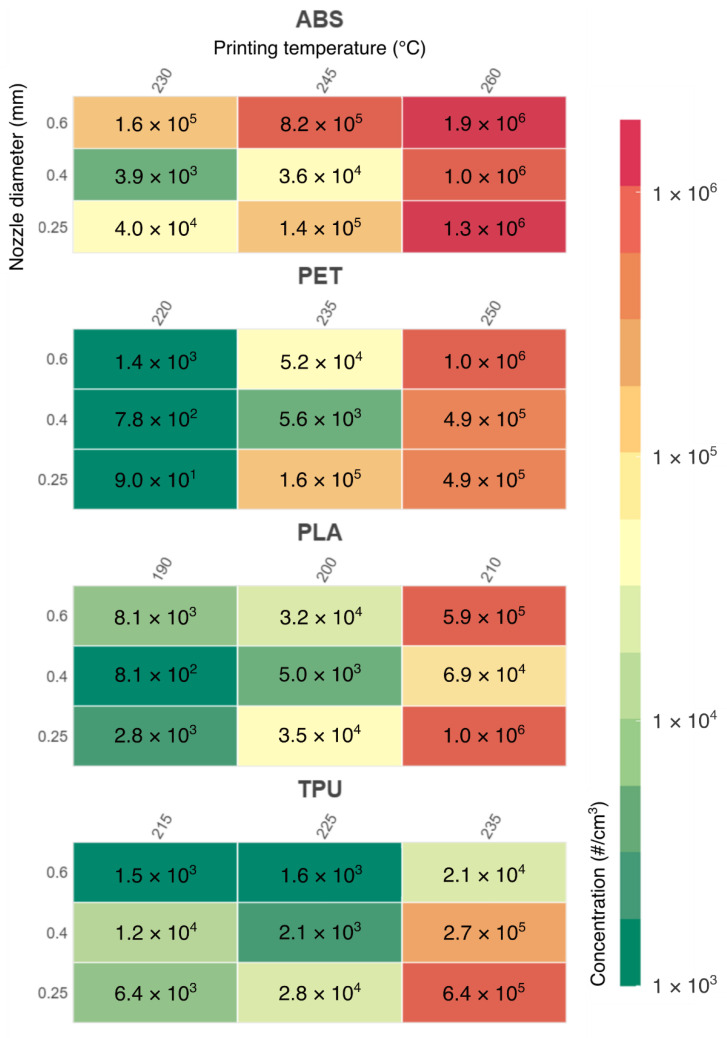
Maximum total particle concentration during the printing process for all measured configurations.

This peak in particle concentration indicates rapid nucleation processes, which occur during the first minutes of printing inside the particle-free chamber. The peak of the distribution was at this moment very sharp, located in the nano-particle range commonly between 8 to 40 nm. Figure 6 shows the maximum total particle concentration for all measured materials, temperatures, and nozzle diameters.

The color bar ranges from 1 × 10^3^ particles/cm^3^ (almost no nucleation) to 1.9 × 10^6^ particles, achieved during the printing of the ABS filament.

After reaching the maximum total concentration of particles, the total concentration usually began to decline slowly, showing the signs of losses caused by particle coagulation, deposition, and air exchange. The growth of the particles was consistently observed and can be seen also in the example in Figure 4. The nucleation of new particles seemed to be suppressed after reaching higher concentrations. An accurate description of the dynamics of this system can be affected by the parameters of our experiment. Undoubtedly, the size of the test chamber and the air exchange rate can have a significant impact.

**Figure 7:**
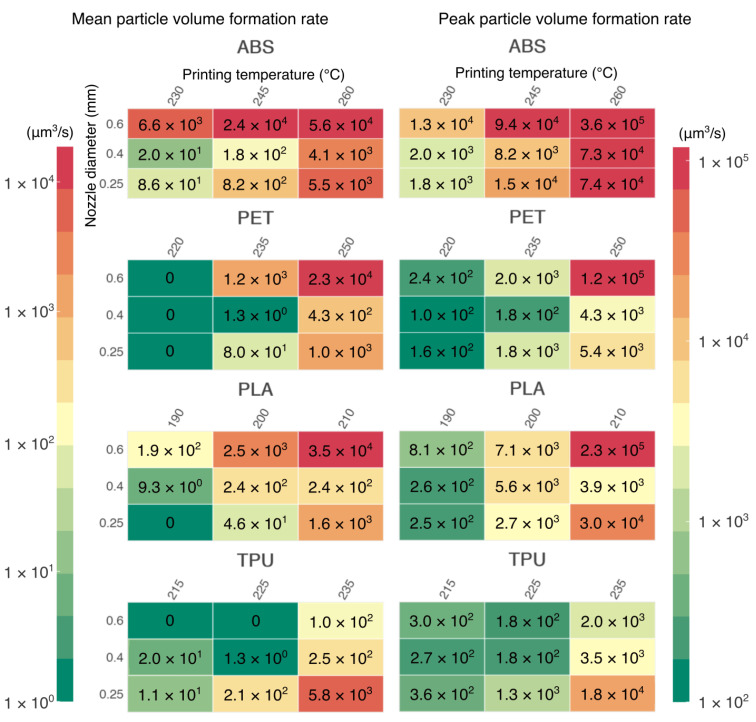
Mean particle volume formation rate during the printing period and peak particle volume formation rate for measured configurations.

Due to the relatively low air exchange rate, and therefore due to intensive coagulation and particle growth, this study chose to evaluate the emissions not by the more common particle number emission rate but by the particle volume formation rate (PVFR). In Figure 7, the mean PVFRs of the whole printing period are presented, as well as the peak PVFR for each configuration. Some of the measured configurations with the lowest emissions resulted in negative mean PVFRs due to some unaccounted particle losses. Those negative values were found for the PET printed at 220 °C, PLA printed at 190 °C with a 0.25 mm nozzle, and TPU at 215 °C and 225 °C with a 0.6 mm nozzle, and they have been fixed to zero in the figure below.

**Figure 8:**
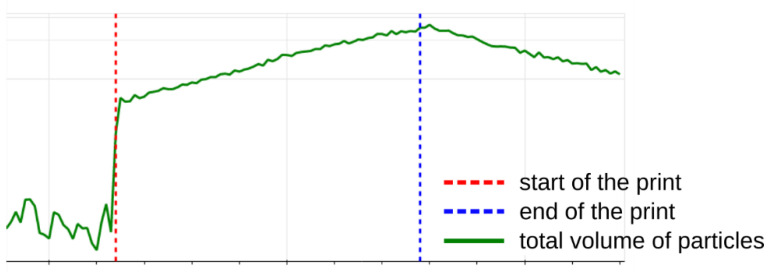
Prevalent progression of the process of particles volume growth during the 3D printing in an enclosed chamber.

Typically, the total volume of particles during the printing was growing or had reached a steady-state value, up to the point when the printing was finished and the printer was not emitting any new particles. The total volume rarely began to decline even before the printing ended. The end of the printing was always followed by a rapid loss of volume of particles (Figure 8).

At the end of the printing period, particle growth stopped and the particle distribution began to decrease. The common range of the peak in concentration in terms of particle diameter at the end was 40–100 nm.

The dependence of particle emissions on printing temperature is very clear. However, it cannot generally be said that emissions will increase with increasing printing temperature. This dependence is always conditioned by the material used. For example, PLA printed at 210 °C may have higher emissions than ABS printed at 245 °C.

The effect of the nozzle diameter on particulate emissions cannot be neglected either. Our research shows that the maximum concentration of FPs does not depend on the mass flow of material or print speed, but rather on the extruder settings. Each of the filament materials tested have optimal extruder settings for which maximum concentrations of FPs can be significantly reduced. For ABS, PLA, and PET, the 0.4 mm nozzle was optimal. For TPU, the larger the nozzle diameter, the lower the emissions. Overall, the best samples in terms of the lowest particle concentrations were PET and PLA printed at the lowest recommended printing temperature regardless of the nozzle. Another evaluation criterion used during the printing was the particle volume formation rate (PVFR), which considers the coagulation and growth of particles in poorly ventilated spaces. This criterion shows similar trends as in the case of maximum concentrations. However, it can be seen that the PVFR is greater for higher extruded material flows and larger nozzle diameters. There is one exception to this and that is TPU again, which has lower emissions while extruded with a nozzle with a larger diameter. 

In the case of a smaller nozzle, the elasticity of the TPU prevents the filament from being pushed correctly through the extruder and maintaining the right tension from the filament feeder. The TPU is then prone to clogging and bubbling inside the smaller nozzle, and this is undesirable both in terms of printing reliability and printing emissions.

## 4. Conclusions

In this article, the impact of printing temperature, extruder nozzle diameter, and filament material on the emissions of FPs was investigated. More than a hundred unified printing tests were performed and analyzed for this study.

Overall, the PLA, PET, and TPU printed at lower printing temperatures had the least emissions. Thus, these configurations can be considered as preferred from a respiratory health perspective. On the opposite side is the ABS material when it is printed at the highest temperature. As for the nozzle, the 0.4 mm nozzle seemed to be optimal for most configurations. An exception was the elastic TPU, which showed lower emissions with a larger nozzle diameter (0.6 mm). Additionally, users should use all sorts of emission protection when printing at higher temperatures and/or with an improper nozzle. We show that such configurations can increase the concentrations of ultrafine particles by three orders of magnitude. 

The most critical period seems to be the beginning of the printing process, when the particles are usually at the highest concentrations and smallest sizes. It is advisable to include sufficient ventilation in the printing rooms or enclose the printer away from humans so that they do not get exposed to these high concentrations. The data obtained here can be used to model the mechanics of particle formation in the future. Because the data contain evolution of the size distribution of the particles over time, it might be possible to model the processes inducing the particle growth.

## Figures and Tables

**Table 1:** Printing combinations, duration of the prints, and the mass flow of filament material during individual prints.

Material	Producer	Color	Printing Temp. (°C)	Nozzle (mm)	Duration (min)	Length (mm)	Mass/min (g/min)
PLA	Fillamentum	Vertigo Grey	190, 200, 210	0.25 0.4 0.6	58 28 20	340 370 380	0.0421 0.0949 0.1364
ABS	Ultimaker	Yellow	230, 245, 260	0.25 0.4 0.6	48 32 22	360 380 400	0.0525 0.0832 0.1275
PETG	Polymaker	Teal	220, 235, 250	0.25 0.4 0.6	49 32 22	340 380 400	0.0547 0.0937 0.1435
TPU	Ultimaker	Red	215, 225, 235	0.25 * 0.4 0.6	60 38 27	360 370 380	0.0476 0.0772 0.1116

* configuration not officially supported.

## Data Availability

Data available in a publicly accessible repository that does not issue DOIs. Publicly available datasets were analyzed in this study. This data can be found here: [https://github.com/RadomirChylek/3d-printing-emission] (access date 1 November 2021).
